# Political influence and air pollution: Evidence from Chinese cities

**DOI:** 10.1016/j.heliyon.2023.e17781

**Published:** 2023-07-03

**Authors:** Wen-Chao Shao, Li-Chen Chou

**Affiliations:** a School of Economics, Nankai University, Tianjin, China; b Department of Applied Economics, Shantou University, Shantou, China

**Keywords:** Political cycle, PM2.5, Chinese city, Chinese communist party congress, Party secretaries

## Abstract

The influence of the political system, and official mobility on the economy, finance, and environment has been confirmed by numerous studies, political cycle may bring a critical impact on the functioning of the economic system. Cities in China change their party secretaries every few years, providing a good context for our analysis. In this study, we analyze the impact of the political cycle on air pollution in 247 Chinese cities from the year 2002–2017. The main results indicate that the Chinese Communist Party Congress bring a significant effect. Air pollution has been significantly reduced in the year before and after the Party Congress, which means the political cycle plays an important role in current Chinese environmental protection or pollution control. In addition, the closer the city is to the capital, the stronger the pollution control, and the city's environmental pollution will be reduced.

## 1 Introduction

China has experienced economic growth for over 40 years since 1979. The rapid economic growth has driven the improvement of China's income per capita, quality of life, and consumption capacity while also has brought about the negative effects of economic development [9]. Among them, the problems of uneven economic development and environmental pollution have become more and more serious along with China's rapid economic growth. Due to the particularity of the political system, China's local governments served as the social administrators of specific geological areas, and economic growth was normally viewed as a key indicator of official promotions in the past [52]. With the increasing participation of the public in environmental governance, the Chinese government has paid more and more attention to environmental pollution [51]. The central government has incessantly promoted the importance of environmental and energy indicators. How to achieve both economic growth and the restraint of environmental pollution at the same time has become an important issue in China's national development in recent years.

There have been many studies on the impact of politics on the economy, environment, and public concern from the aspects of the impact of the political institutions [7,13,14,24,30,35,37], fluctuations in currency exchange rates [5,16,27], educational institutions and financial budget [1,11,40], environmental impact (Nieet al., 2013), environmental regulations, energy efficiency and consumption [12,22], etc. In China, the impact of the political cycle is quite comprehensive. Uncertainty from official turnover could have a significant environmental impact [3]. However, few studies have discussed the cyclical fluctuations of environmental regulations and their performance in the context of the political cycle in China on the issues of environmental pollution.

At present, the Chinese government divides cities into municipalities directly under the county-level cities, prefecture-level cities, sub-provincial cities, and central government, then appointed a municipal party secretary as the government representative in each city. A municipal party secretary is in charge of the comprehensive party and government work in the city, organize the activities of the standing committee of the municipal party committee, responsible for the city work of the municipal party committee mainly. The difference between European countries and America lies in that those countries do not have an affiliate or governing organization of a political party as China does in the administrative units of cities [40]. In China, a municipal party secretary is the actual ruler of the city while the major is the person in charge of the administrative affairs. Moreover, China's Communist Party holds national party conventions every five years, carries out a series of appointments and dismissals of local administrators at the same time, and drafts the government policy for the next phase. Therefore, the changing of local administrators directly or indirectly influences the carry-out and planning of policies in the economy, finance, environment, etc., both currently and in the future. In certain events and situations (such as years when there was the 2008 Beijing Olympic Games, the 70th anniversary of the National Day in 2015 after World War II, and the 2017 G^20^ Summit), local governments even took comprehensive environmental governing measures such as traffic control, supervision over enterprises shutdown and requirements of the urban people and townspeople to travel outside to achieve” Political Blue Sky” [38]. Therefore, China's political cycle is an important variable that affects the state of the environment and the quality of life.

From Figgure 1, which has sorted out the changes in China's smog pollution over the years and the surface concentration of PM 2.5, the tendency of improvement in China's smog pollution since 2007 can be seen. Viewed by regions, the data show that central provinces have seen the worst pollution while western areas have seen the lightest. Differences in the scale of pollution are mainly related to the economic structures of China's geological regions. In the economic development of the coastal and eastern provinces over the past 40 years, the main economic activities have shifted gradually from manufacturing, and industry to service-oriented businesses while the production base of manufacturing has been transferred to the central and western regions [9]. Viewed by years, the data show that the state of pollution became better one year after the 17th, and 18th Party Congress. There has been a particular improvement in the state of pollution since 2012. Historical data viewed, the state of pollution in China's cities truly improved after each Party Congress between 2003 and 2013. Whether the environmental governance is influenced by the political cycle? If so, to what extent is it influenced? This study focuses on the analysis and discussion of these questions through the collection of empirical, including the economic data variables of 247 Chinese cities from 2002 to 2017, the annual average concentration of 2.5 μm of fine particulate matter (PM2.5) in the air, records of the change of party secretaries in each city, and calls to Party congresses in China. The main results, also the empirical results in the study, show that the overall Chinese cities' air pollution worsened two years before and after the Party Congress while improved one year before and after the Party Congress. The contribution of this paper is to study the influence of the political cycle on cities' air pollution by taking the Party Congress as a political cycle and analyzing the influence of cities' party secretaries' personal characteristics. The structure of the study is as below consisting of the second part reviewing the literature, the third part showing the methodology and empirical data, the fourth part containing the empirical result, and the last part concluding the study.

## 2 Literature review

The quality of the living environment has an important impact on people's health. The low quality of the living environment may cause the growth of bacteria and viruses, causing personal discomfort or the spread of infectious diseases; it may also cause pollution problems, which in turn affect the use of natural resources [[19], [20], [21],^39^]. Previous literature concluded that changes in environmental quality have critical effects on individual health, mortality, or disease transmission. Health economics and other related studies have pointed out that the improvement of environmental conditions has a significant positive effect on personal health, especially the reduction of infant mortality [15,17,18]. However, this series of discussions emphasize the short-term impact of environmental conditions on the currently explained variables and does not discuss whether there are cyclical changes or impacts.

The change in the economic environment is of significance to the development of a country and the economy. It has been extensively studied in the literature. In studies on the political factors that influence economic development, the literature mainly focuses on factors like state institutions, government policy, and corruption level, etc. [31] pointed out that political institutions and government change had a key impact on the economic environment. The setting of the institutions could reduce the uncertainties and transaction costs. Corporate behavior in the economic society was not only affected by official institutions but also regulated by non-official institutions [32,44]. [2] studied the relationship between politics and economic growth in a simple model of endogenous growth, and concluded that maximizing growth is optimal only for a government that cares solely about pure “capitalists.” The greater the inequality of wealth and income, the higher the rate of taxation, and the lower growth [4]. examined the effect of the “quality” of the institutional framework on economic development and the results indicated that economic development requires not only physical and human capital formation but also a freedom to choose and institutional support [36]. concluded that the influence of the quality of institutions was larger than other factors such as geography, trade, income levels, etc. [6] construct indices of economic policy uncertainty based on newspaper coverage frequency. The results found that an intense presidential election (commonly seen when the ruling party switches sides) exerted no smaller influence on economic policy uncertainty than a major crisis and such instability brought out by regime change tended to be more and more severe in the near future.

The most important thing in the political system is officials, and the performance of officials in office is also the embodiment of the political system. Some Chinese researchers focus on the study of the impact of official replacement on the economy [42,43]. concluded that when local government officials in China change, the newly appointed local officials tend to invest in productive public goods to achieve new economic performance in new positions and often enhance economic growth performance. In this way, local officials will promote economic growth through economic activities and thus gain political promotion, but it will lead to problems such as fiscal imbalance and budget deficit, which will lead the government to raise funds by leasing public resources [48]. conducted an empirical study on the sample of official changes in prefecture-level cities from 1999 to 2013 and found that policy factors and policy risks in China's economic growth were significantly weakened due to the weakening of economic evaluation indicators by the government in recent years [42]. explored the impact of various local officials' replacements on the short-term economic growth system. They used the sample data of officials' replacement in 29 provinces and autonomous regions in China from 1979 to 2006 to test and found that the replacement of governors and provincial Party secretaries would have a short-term negative impact on economic growth. And its influence degree is different with the change frequency of local officials, the age of officials, and other factors. The replacement of local officials will only affect short-term economic fluctuations, not long-term economic growth trends. So who decides the change of local officials in China? In China, the appointment and removal of officials are in the hands of higher party organizations, the two sessions and the Conference of Party Representatives of the Communist Party of China are held every five years, which have become China's regular political cycle meetings.

In the studies on how China's political institutions affect the environment, the past literature has pointed out that the change and uncertainty of the Chinese government led to the uncertainty of the following economic policy and implementation strategy [33,34], thus further affected the unity between local government and enterprises and the fluctuation of the asset value of enterprises in the market [28]. China has annually convened the National People's Congress and the Political Consultative Conference (referred to as the two sessions) to decide the allocation and promotion of the next annual policy budget since 1959. The two sessions and the Conference of Party Representatives of the Communist Party of China held every five years thus have become China's regular political cycle meetings [10]. through empirical study found a significant relation between “the two sessions” cycle (changes in central government) and the economic fluctuation cycle. Changes in central government generated communication cycles of local governments. The promotion mechanism under the assessment of economic performance induced vertical and horizontal competition among local governments and produced periodic economic development measures thus increasing the amplitude of the economic cycle. A study on the effects of the political cycle on coal mining accidents by Ref. [29] found that the government sought to stabilize society by controlling coal mine output rather than strengthening the safety measures and there were big differences due to varying degrees of media attention from place to place. [8]; and [47] found that investments from enterprises decreased significantly when faced with political uncertainties like changes of local officials. And political uncertainties also weakened the credit resource advantage of politically related enterprises, thus affecting investment spending [48]. conducted an empirical study of the sample of changes in prefecture-level city officials from 1999 to 2013 and found that as the government weakened the economic assessment indicator in recent years, policy factors and policy risks in China's economic growth were significantly weakened.

Since China implements a system of political concentration and economic decentralization [46], the central government took into consideration the efficiency of the local policy implementation and economic performance, etc., when assessing the performance of local governments. In the phase of reform and opening that started in the 1980s, in the political context with economic construction as the core, infrastructure, and GDP increasing rate played more important roles than environmental protection in political performance assessment. Therefore, [42,43]; and [45] pointed out in the study that when officials changed in China's local governments, the newly appointed local official out of consideration of political promotion normally tended to reduce the intensity of environmental governance to usher in industries with high energy consumption, high pollution, and high emissions as well as to invest in productive public goods to strengthen the performance of economic growth. This type of economic construction features an advantage of a short investment cycle and rapidly promotes local economic development. While in contrast, benefits of consumer public investment such as spending on environmental protection usually have a cycle of longer periods, and thus are not favored by officials. As a result, local officials carried out economic activities to promote economic growth for political promotions resulting in problems as high pollution and environmental quality deterioration. Although the Chinese government has emphasized that officials' performance should include environmental protection work since 2005, however, it was not until the implementation of the Air Pollution Prevention and Control Action Plan of the State Council of China in 2013, and the increasing pressure from the central government and the public on local governments to strengthen environmental protection that environmental protection work gradually became the assessment basis that affected the promotion of local officials [50]. [25] concluded that by giving the visibility of policy, if the objectives of environmental performance assessment are clearly defined, the responsibility is in place, and the public has high visibility, the pollution control effect will bring a high-quality outcome. And if the visibility is low, although the environmental protection assessment is included, the pollution control effect will bring low efficiency.

## 3 Methodology

According to Refs. [26,49]; this study analyzes the dynamic problem of environmental pollution by introducing investment and assumes that the benefits brought to local government officials through capital stock ($K_{t}$) and investment ($I_{t}$) are as following:

(1) $$Y_{t}=AK_{t}$$

$Y_{t}$ is the local economic output value; A is a constant greater than 0 and represents the contribution of investment to GDP growth. The investment of local officials in period t is $I_{t}$, and since $I_{t}$ needs to pay interest costs, here cost function is denoted as $C\left(I_{t}\right)$, $C^{\prime }\left(∙\right)>0,C^{\prime \prime }\left(∙\right)>0$. Due to the depreciation problem of fixed assets, δ is forthe capital depreciation rate, and the capital stock at t+1 period is:

(2) $$K_{t+1}=\left(1-\delta \right)K_{t}+I_{t}$$

At the same time, environmental pollution problems will occur after an investment is transformed into production. Under the condition of the unchanged environmental treatment technology, 1 unit of investment produces corresponding q units of pollutants, and the cost of environmental pollution per unit of investment is q. The economic income generated by the capital stock $K_{t}$ is denoted as $V\left(K_{t}\right)$:

(3) $$V\left(K_{t}\right)=\max_{I_{t}}\left\{AK_{t}-C\left(I_{t}\right)+\beta V\left[K_{t+1}\left(1-q\right)\right]\right\},0<\beta <1$$

Sincethe promotion in local government is associated with economic growth [52], local officials have incentives to maximize economic benefits through investment. Therefore, the optimal dynamic condition is that local officials maximize equation (3) through $I_{t}$ under the condition of equation (1), and the environmental pollution corresponding to $I_{t}$ is $I_{t}q$.

Due to the uncertainties from the government, future benefits for officials are uncertain. Parameter β in Equation (3) can be regarded as officials' evaluation of the future political environment. The smaller β is, the lower the officials’ evaluation of future revenue is. The Party Congress cycle changes β. We use $\beta^{0},\beta^{1},\beta^{2}$ to represent the motivation levels of local officials' investment at the last party congress held in the year in the middle of the two Party Congresses and in the time when the next Party Congress will be held, respectively, which meets $\beta^{0}>\beta^{1}>\beta^{2}$.

The uncertainties right after the Party Congress are relatively low and increase as it draws closer to the next Party Congress. The Euler equation for the optimal dynamic condition for local officials is:

(4) $$\dot{C}\left(I_{t}\right)=\beta^{i}\left[\dot{C}\left(I_{t+1}\right)+A\right]$$

i = 0,1,2. The left side of the equation represents the marginal cost of the investment in period t and the right side represents the marginal incomes. According to the Euler equation, the followings can be obtained:

(5) $$\dot{C}\left(I^{0}\right)=\frac{\beta^{0}\left(1+\beta^{1}+\beta^{1}\beta^{2}\right)}{\left(1-\beta^{0}\beta^{1}\beta^{2}\right)}A$$

(6) $$\dot{C}\left(I^{1}\right)=\frac{\beta^{1}\left(1+\beta^{2}+\beta^{0}\beta^{2}\right)}{\left(1-\beta^{0}\beta^{1}\beta^{2}\right)}A$$

(7) $$\dot{C}\left(I^{2}\right)=\frac{\beta^{2}\left(1+\beta^{1}+\beta^{0}\beta^{1}\right)}{\left(1-\beta^{0}\beta^{1}\beta^{2}\right)}A$$

$I^{0}$, $I^{1}$, $I^{2}$ in equations (5), (6), (7) represent the investment conditions of local officials in the year when the last Party Congress was held, in the middle of the two Party Congresses and the time when the next Party Congress was held. Local government officials have different levels of motivation in developing the local economy and different levels of investment at different periods of the political cycle. Since $C^{\prime \prime }\left(∙\right)>0$, $\beta^{0}>\beta^{1}>\beta^{2}$, we obtain that $I^{0}>I^{1}>I^{2}$. Presumably, after the party congress, local government officials invested more in economic development, resulting in more environmental pollution. The closer to the next Party Congress during the two congresses, because of the more political uncertainties, the less incentive for officials to invest in the economy, thus the less environmental pollution generated. Equations (5), (6), (7) show that environmental pollution in China is correlated with the political cycle.

Accordingly, the empirical estimated formula of this paper is set as below:

(8) $$\log\left(PM2.5_{it+1}\right)=\alpha +\sum \beta_{i}X_{it}+\sum \gamma_{j}{Conference}_{jt}+\sum \beta_{k}S_{kt}+\varepsilon_{it}$$

$PM2.5_{it+1}$ is the air condition of the city in the coming year. $X_{it}$ is the socioeconomic variable of the current year, ${Conference}_{jt}$ is the dummy variable reflecting whether the Party Congress will be held or not and $S_{kt}$ is the personal characteristic of the municipal party secretary.

## 4 Data and empirical results

The empirical data in this paper mainly come from three aspects. The variables Capita, Finance Pressure, and Wage are from the 2002–2017 China City Statistical Yearbook. The data publishes a number of survey statistics on urban environmental protection, economy, population, etc., with more than 250,000 people engaged in non-agricultural industries in the urban area every year. The annual average concentration of fine particulate matter (PM2.5) in the air is derived from the 1998–2016 global historical PM2.5 annual average data set released by Columbia University International Geoscience Information Network Center (see http://sedac.ciesin.columbia.edu). The variable PM2.5 refers to particles with a diameter of 2.5 μm or less (less than 1/20 of the thickness of a human hair), also known as inhalable lung particles. The higher the PM2.5 value, the poorer the air quality and the higher the health risks people face in the city. Secondly, variables such as whether the Party Congress was held in the year (Conference), whether there's a change of the city secretary in each city (Change), the age of the city secretary (Age), education level of the secretary (variable Education is the value that can be 0, 1, 2, 3 or 4 with each value indicating that the education level of the secretary is other, primary school, high school, university or postgraduate), Term of office (Term) comes from databases of the Chinese party and government leaders published by Baidu Encyclopedia, Wikipedia and the Communist Party of China News Network (see http://cpc.people.com.cn/index.html). Since the Chinese government mostly gives brief descriptions of officials' personal information, it causes difficulties in data construction. For example, when officials attended school or when they obtained their highest education degree is not disclosed on government websites. Therefore, we can only set category variables for statistics.

Besides, the literature points out that the further away from the central government, the greater the discretionary power the local government has. If the local government takes economic growth as its main goal, the possibility of environmental pollution and damage is higher. The distance between the central government or provincial government and local governments reflects the level of supervision costs. When the supervision cost gets higher, it is more difficult for the government to control the environmental pollution between regions [41]. To analyze whether the supervision of a city by the central or provincial government will affect its air quality, the variables Distance1 and Distance2 take into account the geographical distance between the city and the capital or provincial capital which is derived from the data of China Transportation Yearbook. In summary, Table 1 sorts out the descriptive statistics and definitions of various variables; Table 2 makes statistics on the frequency of replacement of the Party Congress and municipal party secretary in China. From the frequency of secretary change, it can be seen that there are more than 150 changes of secretary each year, and the number of changes was the greatest in the current year and the following year of the party congress.

Table 3 analyze the data including all provinces. The outcomes in the two tables are the estimated results of fixed effects under the control of different variables. In the result of FE1, we do not control any other variables and only take the time from the Party Congress as the explanatory variable. The results show that the time to the Party Congress bring significant at the level of 1%, indicating that the closer the Party Congress is held, the higher the degree of the environmental pollution.

In the estimation of FE2, we controlled variables such as per capita output (log (Capita)), financial pressure (Finance Pressure), labor wages (log (Wage)), and whether the secretary was changed (Change). We believe that as the Party Congress is approaching, due to the uncertainty of the government, the future earnings are uncertain for the incumbent secretary. Therefore, the enthusiasm of local officials to invest is not high, and the degree of environmental pollution has slowed down. After the end of the Party Congress, the current secretary increased investment due to performance considerations, which in turn led to increased environmental pollution, manifested in the increase in environmental pollution in the second year after the end of the Party Congress. The regression results show that the time to the Party Congress is always significant at the 1% level. However, after adding the new variable, there has been a major change, that is, the city and environmental pollution are negatively correlated one year before and after the party congress. This is consistent with our assumption.

In the estimation of FE3, we control the personal characteristics and changes of the city secretary. Adding the two variables of education level and age as control variables, we believe that the cognition and education level of the secretary is related to their environmental protection awareness. The regression results show that the time to the Party Congress is still significant at the level of 1%, which shows that air pollution has been significantly reduced in the year before and after the Party Congress. Among them, the age and education of the secretary are significant at the level of 1%. It can be seen that as the age of the secretary increases, the number of years of education is higher, and the degree of environmental pollution decreases. The change of the secretary is only significant at the level of 10%, and the length of his tenure is not significant.

Finally, in the estimation of FE4, the results indicate that the time to the Party Congress is still significant at the level of 1%. The city and environmental pollution in the year before and after the party congress are negatively correlated, and the city and environmental pollution in the two years before and after the party congress are positively correlated. The changes brought about by the new variables are the same as FE2, which once again strongly confirms that the education level and age of the secretary have a negative impact significantly on environmental pollution. Moreover, with the addition of variables, the R-square of the estimation increase.

According to Ref. [41]; the cost of government supervision of a city mainly lies in the distance between the central government and local governments from the city. The closer the distance, the smaller the supervision cost or the stronger the political control. Do the cities closer to the provincial capital or capital lower than that in remote cities due to government supervision? In Table 4, we further consider the distance variable in the estimations. We adjust the OLS estimation of equation (8) and set it as:

(9) $$\log\left(PM2.5_{it+1}\right)=\alpha +\sum \beta_{l}{Distance}_{i}+\sum \beta_{i}X_{it}+\sum \gamma_{j}{Conference}_{jt}+\sum \beta_{k}S_{kt}+\varepsilon_{it}$$

Variable Distance indicates the straight-line distance between the city and the provincial capital (or the capital, i.e., Beijing). We believe that the government's supervision of each city can affect the environmental pollution situation. Therefore, in the estimation of FE5 and FE6, the distance from the city where the secretary is located to the capital Beijing and the distance from the city where the secretary is located to the provincial capital are controlled respectively, which are used as the central supervision cost and the local supervision cost.

In the result of FE5, it can be seen that the time to the Party Congress is still significant at the level of 1%, and the newly added variables are not significant. The outcome indicates that the central government's supervision of local governments has little impact on the decision-making of the secretary in cities. According to the results of FE6, variable log (Distance2) has a positive significant effect, which means the pollution of the remote cities larger than the cities near the capital, indicating that the closer the city is to the provincial capital, the stronger the pollution control and the city's environmental pollution will be reduced.

In the estimation of FE7, we controlled each variable except for the characters of the party secretary. The result shows that one year before and after the Party Congress is significant at the 1% level and brings a negative effect on the scale of PM2.5, the distance variables also indicate that government supervision has a strong supervisory effect on environmental pollution. Finally, FE8 controlled the variables of the secretary's individual character, the empirical result consists of the outcome of FE5-FE7. Table 4 also shows that as we add more variables to the estimation, the R-square of the estimation increase, indicating that the explanatory ability increase in the estimations.

According to Ref. [49]; older secretaries are approaching retirement, their governance is more conservative, and their motivation to carry out economic construction in their cities is lower. Considering that the Chinese government has paid more and more attention to environmental governance in recent years, will there be differences in the implementation of environmental policies by different age groups? In other words, in the context of emphasizing environmental protection, will the secretary close to retirement be more conservative in the implementation of pollution prevention and control? Therefore, Table 5 explores whether the political cycle has different changes in air pollution under different age groups. The empirical results show that the year before and after the party congress has a higher impact on urban pollution reduction for secretaries younger than 55 years old than other groups, showing that the age of the ruling party is also a key factor affecting urban pollution prevention. Furtherly we also separate the samples in coastal and non-coastal regions, the results are also consistent with previous estimations that in one year before or after the Party Congress, air pollution was reduced significantly.

In summary, the empirical results show that air pollution reduced significantly in the year before and after the party congress, showing a political cycle. Combined with the above analysis, we believe a possible reason to explain the phenomenon: On the eve of the Party Congress, political uncertainty was high, which affected the incentive of government officials. From the point of view of officials, the expected impact of political uncertainty in the convening of the party congress reduced their valuation of the future benefits from the economic production process, thereby reducing economic activities. After the convening of the Party Congress, political uncertainty has decreased. Local officials have incentives to accumulate political achievements for their own promotion path by attracting investment and promoting regional economic growth. The main result in our study is similar to Ref. [23]; the authors found that due to the uncertainty in the election results of government officials, companies will reduce investment in election years and return to normal in non-election years. The difference between this article and the other literature is that, compared with countries where there is a clear local public office election year, the change of public officials in China is not affected by the general election system. Therefore, the Party Congress has become an indicator for analyzing political uncertainty and environmental quality change.

## 5 Conclusion

We apply the empirical model to estimate the influence of the political cycle on air pollution in 247 Chinese city samples from the year 2002–2017, we found that the air quality improved in the year before and after the Party Congress and deteriorate in others. Our findings on the political cycle of air pollution also indicate that cities with party secretaries over the age of 55 are statistically significantly less polluted than others. In addition, from the perspective of Chinese economic development in the past 40 years, the economic development of China's coastal areas is higher than that of non-coastal areas, and the government's land and public resources utilization costs are higher. Empirical outcomes indicate that the party congress has a greater impact on PM2.5 in coastal areas than in other areas, which may be a long-term reflection of regional economic performance differences.

Compare with each empirical result and we conclude: 1. China's environmental pollution has shown improvement in the year before and after the Party Congress, and has shown a trend of deterioration in the two years before and after the Party Congress. 2. China's environmental pollution problem is affected by government regulation, especially local government regulation. The farther away from the capital and provincial capitals, the higher the cost of government supervision. The secretary's decision-making is less supervised, and environmental pollution is more serious than in cities close to the capital and provincial capital. According to Refs. [40,49]; Chinese officials have incentives to stimulate economic performance through the transfer of land or public resources in the vicinity of the Chinese Communist Party Congress, thereby consolidating their positions. The research in this article shows that as environmental issues have received increasing attention from the Chinese government and the general public, the city's governing secretaries also have similar behaviors in environmental governance or pollution prevention.

The findings of this study allow us to judge whether China's current or future environmental pollution control or related policy promotion will be implemented smoothly, based on the cycle of environmental pollution fluctuations in statistical data. We can assess whether China's overall environmental pollution will improve or worsen based on whether the Party Congress is approaching in that year. The results of this paper help us better understand the impact of political cycles on environmental pollution, and also bring policy implications. The empirical results reflect that there is a relationship between local economic activities and government change, which in turn affects the degree of environmental pollution. Since the 1980s, the Chinese government's pursuit of economic growth has resulted in a trade-off between environmental quality and economic activity. If the promotion mechanism for local officials does not only focus on economic development, but includes more factors such as education, environmental governance, social security and other public services in the assessment, optimizing the incentive mechanism for officials, maybe to improve environmental pollution, improve A long-term measure of quality of life for residents. Finally, through this study, it can be found that the political cycle has an impact on the operation of China's environment. However, the lack of officials' individual characteristics limits the depth of our research. For example, we can not further analyze whether the cultural norm affects officials' policy implementation due to the data on the government website does not disclose more detailed information. If a more complete construction can be carried out on the data of local officials, future research will be able to expand the analysis of the political influence on China's urban land, finance, labor, and other issues.

## Author contribution statement

Wen-Chao Shao: Performed the experiments; Wrote the paper.

Li-Chen Chou: Conceived and designed the experiments; Analyzed and interpreted the data; Contributed reagents, materials, analysis tools or data; Wrote the paper.

## Data availability statement

Data will be made available on request.

## Additional information

No additional information is available for this paper.

## Declaration of competing interest

The authors declare that they have no known competing financial interests or personal relationships that could have appeared to influence the work reported in this paper.

## Figures and Tables

**Fig. 1:**
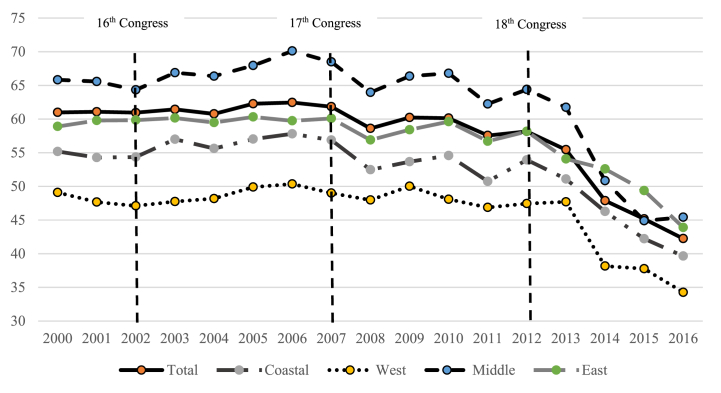
The evolution trend of China's smog pollution. Unit: PM2.5 mean surface concentration (ug/m^3^).

**Table 1:** Descriptive statistics

Variable	Mean	Observations	Definition
PM2.5	57.486	5253	The annual average concentration of particles with a diameter of 2.5 μm or less (less than 1/20 of the thickness of a human hair)
Capita	189,851	4756	Output per capita. (Unit: RMB)
Finance Pressure	2.638	4501	Fiscal expenditure is divided by fiscal revenue. (Unit: 10,000 RMB)
Wage	31,378	4772	Average annual wage of employed persons. (Unit:RMB)
Change	0.244	5615	Dummy, if the party secretary changed in the year = 1, others = 0
Conference	0.176	5661	Dummy, if the Party congress held in the year = 1, others = 0
Distance1	1526.719	5627	The straight-line distance between the city and the provincial capital (unit: kilometers)
Distance2	289.832	5202	The straight-line distance between the city and the capital (unit: kilometers)

Source: This study, [40].

**Table 2:** The statistics of the Party congress and the characters of party secretary

The statistics of the Communist Party Congress	Number of changes of city's party secretary	Ratio (%)	
In the second year before the party congress (Conference t-2)	212	16.49	
In the first year before the party congress (Conference t-1)	254	19.75	
In the year the party congress was held (Conference t)	280	21.77	
In the first year after the partycongress (Conference t+1)	384	29.86	
In the second year after the party congress (Conference t+2)	156	12.13	
Total	1286	100	
Characters of Party Secretary	Mean	Min	Max
Age	52.008	30	69
Education Level	2.766	0	4
Term	2.707	0	12

Source: This study, [40].

**Table 3:** Results 1

Dependent Variable	log (PM2.5)			
	(1)	(2)	(3)	(4)
	FE1	FE2	FE3	FE4
log (Capita)			0.036***	0.037***
			(0.001)	(0.002)
Finance Pressure			0.007***	0.008***
			(0.002)	(0.002)
Conference t-2	0.252***	0.231***	0.070***	0.071***
	(0.008)	(0.008)	(0.009)	(0.009)
Conference t-1	0.277***	0.272***	−0.048****	−0.047***
	(0.010)	(0.010)	(0.015)	(0.015)
Conference t	0.356***	0.346***	0.056***	0.057***
	(0.010)	(0.010)	(0.013)	(0.013)
Conference t+1	0.256***	0.252***	−0.069***	−0.068***
	(0.010)	(0.040)	(0.015)	(0.016)
Conference t+2	0.334***	0.315***	0.053***	0.055***
	(0.010)	(0.010)	(0.013))	(0.013)
Change		−0.009*	0.004	0.005
		(0.005)	(0.004)	(0.005)
log (Wage)			0.001	0.004
			(0.008)	(0.008)
Education Level		−0.023***		−0.008***
		(0.003)		(0.003)
Term		−0.001		0.002
		(0.001)		(0.002)
Age		−0.004***		−3.20E-5
		(0.001)		(0.001)
Year Dummies	Yes	Yes	Yes	Yes
Constant	Yes	Yes	Yes	Yes
N	5253	5044	4418	4340
R square	0.371	0.396	0.549	0.550

Standard errors in parentheses, *p < 0.1, **p < 0.05, ***p < 0.01.

**Table 4:** Results 2

Dependent Variable	log (PM2.5)			
	(1)	(2)	(3)	(4)
	FE5	FE6	FE7	FE8
log (Distance1)	0.002		0.005*	0.005*
	(0.005)		(0.003)	(0.003)
log (Distance2)		0.005**	−0.001	0.001
		(0.003)	(0.006)	(0.006)
Conference t-2	0.249***	0.248***	0.072***	0.073***
	(0.008)	(0.008)	(0.009)	(0.009)
Conference t-1	0.277***	0.274***	−0.046***	−0.044***
	(0.010)	(0.010)	(0.015)	(0.016)
Conference t	0.356***	0.354***	0.059***	0.060***
	(0.009)	(0.010)	(0.013)	(0.014)
Conference t+1	0.256***	0.250***	−0.070****	−0.068***
	(0.010)	(0.010)	(0.016)	(0.016)
Conference t+2	0.333***	0.333**	0.058***	0.059***
	(0.010)	(0.011)	(0.013)	(0.013)
Characters of Party Secretary	No	No	No	Yes
Control Variables	No	No	Yes	Yes
Year Dummies	Yes	Yes	Yes	Yes
Constant	Yes	Yes	Yes	Yes
N	5177	4785	4046	3971
R square	0.373	0.375	0.544	0.544

Standard errors in parentheses, *p < 0.1, **p < 0.05, ***p < 0.01.

**Table 5:** Results 3

Dependent Variable	log (PM2.5)			
	(1)	(2)	(3)	(4)
	Age<55	Age≧55	Coastal	Non-Coastal
log (Distance1)	0.002	0.007	0.004	0.003
	(0.003)	(0.005)	(0.004)	(0.003)
log (Distance2)	0.013*	−0.033***	0.002	−0.001
	(0.007)	(0.011)	(0.009)	(0.007)
Conference t-2	0.069***	0.056***	0.060***	0.078***
	(0.012)	(0.015)	(0.015)	(0.012)
Conference t-1	−0.041**	−0.037	−0.122***	−0.011
	(0.019)	(0.033)	(0.024)	(0.020)
Conference t	0.059***	0.067***	0.045**	0.064***
	(0.017)	(0.025)	(0.022)	(0.017)
Conference t+1	−0.078***	−0.032	−0.092***	−0.060***
	(0.020)	(0.036)	(0.025)	(0.021)
Conference t+2	0.051***	0.048*	0.041*	0.064***
	(0.016)	(0.023)	(0.021)	(0.017)
Characters of Party Secretary	Yes	Yes	Yes	Yes
Control Variables	Yes	Yes	Yes	Yes
Year Dummies	Yes	Yes	Yes	Yes
Constant	Yes	Yes	Yes	Yes
N	2800	1171	1248	2723
R square	0.501	0.635	0.569	0.550

Standard errors in parentheses, *p < 0.1, **p < 0.05, ***p < 0.01.
